# Investigation of the causal relationship between ALS and autoimmune disorders: a Mendelian randomization study

**DOI:** 10.1186/s12916-022-02578-9

**Published:** 2022-11-02

**Authors:** Paria Alipour, Konstantin Senkevich, Jay P. Ross, Dan Spiegelman, Despoina Manousaki, Patrick A. Dion, Guy A. Rouleau

**Affiliations:** 1 Montreal Neurological Institute and Hospital, McGill University, Montréal, QC Canada; 2 Department of Human Genetics, McGill University, Montréal, QC Canada; 3 Department of Neurology and Neurosurgery, McGill University, Montréal, QC Canada; 4 Department of Biochemistry and Molecular Medicine, University of Montreal, Montréal, QC Canada; 5 Department of Pediatrics, University of Montreal, Montréal, QC Canada; 6 Research Center of the Sainte-Justine University Hospital, Montréal, QC Canada

**Keywords:** Mendelian randomization, Amyotrophic lateral sclerosis, Autoimmune disorders, Causal relationship

## Abstract

**Background:**

Epidemiological studies have reported an association between amyotrophic lateral sclerosis (ALS) and different autoimmune disorders. This study aims to explore the causal relationship between autoimmune disorders and ALS using Mendelian randomization (MR).

**Methods:**

To test the genetically predicted effects of liability towards immune-related outcomes on ALS risk, we used summary statistics from the largest European genome-wide association studies (GWAS) for these disorders in a two-sample MR setting. To do this, we extracted single nucleotide polymorphisms (SNPs) from the GWAS, which strongly associated with the 12 traits, and queried their effects in a large European ALS GWAS (27,265 cases and 110,881 controls). To avoid bias in our MR instruments related to the complex linkage disequilibrium structure of the human leukocyte antigen (HLA) region, we excluded SNPs within this region from the analyses. We computed inverse-variance weighted (IVW) MR estimates and undertook sensitivity analyses using MR methods robust to horizontal pleiotropy. We also performed a reverse MR analysis testing the causal effects of ALS on the above autoimmune traits.

**Results:**

After applying Bonferroni correction for multiple testing, our MR analyses showed that the liability to autoimmune disorders does not affect ALS risk. Our reverse MR analysis also did not support the effects of liability to ALS on other autoimmune disorders. The results of the main IVW MR analyses were generally supported by our sensitivity MR analyses. The variance in the exposures explained by the sets of SNPs used as MR instruments ranged from 8.1 × 10^−4^ to 0.31. Our MR study was well-powered to detect effects as small as an odds ratio (OR) of 1.045 for ALS in the main MR and as small as an OR of 1.32 in the reverse MR.

**Conclusion:**

Our MR study does not support a relationship between liability to autoimmune disorders and ALS risk in the European population. The associations observed in epidemiological studies could be partly attributed to shared biology or environmental confounders.

**Supplementary Information:**

The online version contains supplementary material available at 10.1186/s12916-022-02578-9.

## Background

Amyotrophic lateral sclerosis (ALS) is a rapidly progressive neurodegenerative disorder that leads to the paralysis of almost all skeletal muscles, with a high mortality rate typically due to respiratory paralysis in the 3–5 years following diagnosis. The lifetime risk of ALS is estimated to be 1 in 400 [1, 2], and less than 10% of patients survive beyond 10 years [3, 4]. ALS is caused by genetic and environmental factors, with both familial and sporadic cases described. The genetic and environmental interplay in ALS varies depending on the innate penetrance of pathogenic variants, polygenic predisposition, and neurotoxicity of specific environmental modifiers [5–7]. Consequently, the identification of underlying pathologies that could trigger ALS might point to avenues for new treatments.

Neuroinflammation and autoimmunity have been reported to be associated with ALS in previous studies [8–16]. The presence of autoimmune disorders has been reported to increase the risk of ALS [17] which could suggest a shared genetic architecture between these diseases. Although epidemiological studies suggest associations between ALS and autoimmune disorders, it remains unknown if these associations are causal. Because of unmeasured confounding and reverse causation, these epidemiological studies are prone to bias hampering the potential for causal inference. Nonetheless, determining if there is a causal association between ALS and autoimmune disorders could point to specific biological pathways and inform prevention strategies.

Mendelian randomization (MR) is a study design that allows to explore the causality between an exposure of interest (here an autoimmune disorder) and an outcome (here ALS) using an instrumental variables (IVs) approach [18]. In MR, genetic variants strongly associated with an exposure, and satisfying specific assumptions are used as IVs to study the causal association with an outcome. Since these variants are randomly assigned at conception, this could reduce bias due to environmental confounders if MR is conducted properly. As such, the MR design is analogous to a randomized controlled trial. In the current study, we applied bidirectional MR to seek evidence of a causal association between autoimmune disorders (celiac disease (CeD), multiple sclerosis (MS), rheumatoid arthritis (RA), Crohn’s disease (CD), psoriasis (PsO), primary sclerosing cholangitis (PSC), asthma, irritable bowel syndrome (IBS), primary biliary cirrhosis (PBC), ulcerative colitis (UC), type 1 diabetes (T1D), systemic lupus erythematosus (SLE)) and ALS using summary statistics from the largest available genome-wide association studies (GWAS) in European populations for the above traits. The term “autoimmune” will be used to describe both autoimmune and autoinflammatory disorders (such as asthma).

## Methods

In order to perform any MR study, the valid genetic variants (SNPs) used as instruments should satisfy three criteria: the relevance assumption—they should be strongly associated with the exposure of interest; the independence assumption—there is no shared common cause with the outcome; and the exclusion restriction assumption—SNPs only affect the outcome through the path of the exposure [19].

### Study cohorts and GWAS

To perform our MR analyses, we used summary-level data from the largest publicly available GWAS for each trait (Table 1). Specifically, the summary statistics of the 12 autoimmune disorders GWAS are available from the GWAS catalog [20, 21] and the IEU OpenGWAS project [22] and the ALS GWAS from the Project MinE [23, 24]. Information on recruitment procedures and diagnostic criteria is detailed in the original publications. All cases and controls in these studies were of European ancestry. Also, there is no significant overlap between GWAS populations.

**Table 1:** Characteristics of the ALS and autoimmune disease GWAS cohorts and results of the power analysis

Disease	Study	Journal	Cases	Controls	Sample size	Min OR MR	Min OR reverse MR
ALS	van Rheenen et al. [23]	Nat. Genet.	27,205	110,881	138,086	–	–
Asthma	Valette et al. [25]	Commun. Biol.	56,167	352,255	408,422	1.700	1.270
CD	M. de Lange et al. [26]	Nat. Genet.	12,194	28,072	40,266	1.260	1.730
CeD	Trynka et al. [27]	Nat. Genet.	12,041	12,228	24,269	1.234	1.190
IBS	Eijsbouts et al. [28]	Nat. Genet.	40,548	293,220	333,768	2.000	1.320
MS	Consortium I.M.S.G., et al. [29]	Science.	47,429	68,374	115,803	1.28	1.510
PBC	Cordell et al. [30]	J. Hepatol.	8021	16,489	24,510	1.190	2.000
PSC	Ji et al. [31]	Nat. Genet.	2871	12,019	14,890	1.180	2.370
PsO	Tsoi et al. [32]	Nat. Genet.	10,588	22,806	33,394	1.045	1.600
RA	Okada et al. [33]	Nature	19,234	61,565	80,799	1.380	1.500
T1D	Forgetta et al. [34]	Diabetes	9266	15,584	24,840	1.180	1.950
UC	M. de Lange et al. [26]	Nat. Genet.	12,366	33,609	45,975	1.290	1.700
SLE	Bentham et al. [35]	Nat. Genet.	5201	9066	14,267	1.440	1.510

*Min OR MR* minimum OR in the main MR for a power of 80%, *Min OR reverse MR* minimum OR in reverse MR for a power of 80%, *CD* Crohn’s disease, *CeD* celiac disease, *IBS* irritable bowel syndrome, *MS* multiple sclerosis, *PBC* primary biliary cirrhosis, *PSC* primary sclerosing cholangitis, *PsO* psoriasis, *RA* rheumatoid arthritis, *T1D* type 1 diabetes, *UC* ulcerative colitis, *SLE* systemic lupus erythematosus

### Mendelian randomization

#### IV selection

For the selection of genetic instruments from each of the eleven exposure GWASs, we used the default settings in the R package TwoSampleMR [36, 37]. Specifically, genome-wide significant (*p*-value < 5.0E−08) SNPs were extracted, and SNPs harboring the HLA region (chr6:27,477,797–34,448,354, hg19/GRCh37) were excluded [38]. For analyses in which there were insufficient IVs (due to SNPs not available in outcome GWAS), we used the LDproxyR tool to replace the IVs with proxy SNPs in high linkage disequilibrium (LD *r*^2^ > 0.8). Standard clumping parameters were used to select independent SNPs (clumping window of 10,000 kb, LD *r*^2^ cutoff 0.001). The proportion of variance of the exposures explained by the SNPs (*R*^2^) and *F*-statistics were calculated to estimate the strength of IVs [39] to satisfy the first MR assumption. The same approach was taken for the reverse MR; as such, we performed 24 bidirectional MR studies, where autoimmune disorders and ALS were regarded either as exposure or as the outcome.

#### Power calculation

Using an online Mendelian randomization power calculation, we set the power to 80% and reported the minimum odds ratio (OR) for each MR analysis [40, 41] (Table 1).

#### Mendelian randomization analyses

Bidirectional two-sample MR was employed in the TwoSample MR R package [36, 37]. After clumping IVs, we performed Steiger filtering to exclude SNPs explaining more variance in the outcome than in the exposure [36]. Then, we applied the inverse variance weighted (IVW) method to combine the effect of different IVs. The Wald ratio was calculated for each SNP, and the individual effect of each SNP was meta-analyzed using IVW to generate the concluding beta estimate, which was transformed to an OR [42–44]. To test the third MR assumption, we applied MR Egger to detect possible violations of instrumental variable assumptions due to directional horizontal pleiotropy [42]. Additionally, we used weighted median (WM) which is a median of the weighted estimates and provides a consistent effect even if 50% of IVs are pleiotropic [45]. Heterogeneity was tested using Cochran’s *Q* test in the IVW and MR-Egger methods [46]. To illustrate the results of the different MR methods, we constructed scatter plots using the TwoSampleMR package. Additionally, we used the MR-PRESSO test to detect outlier SNPs which may be biasing estimates through horizontal pleiotropy and adjust for these [47]. Finally, a leave-one-out analysis (LOO) was performed to detect if there is any single SNP disproportionately responsible for the result of each MR study.

### Multivariable MR

UC and CD have been reported to have a genetic association [48]. To control for the pleiotropic effect in this study, we run multivariable MR [49] for the studied traits (UC, and CD). The significant SNPs were extracted, then combined and clumped. The analysis was performed using the TwoSampleMR R package.

## Results

**Fig. 1:**
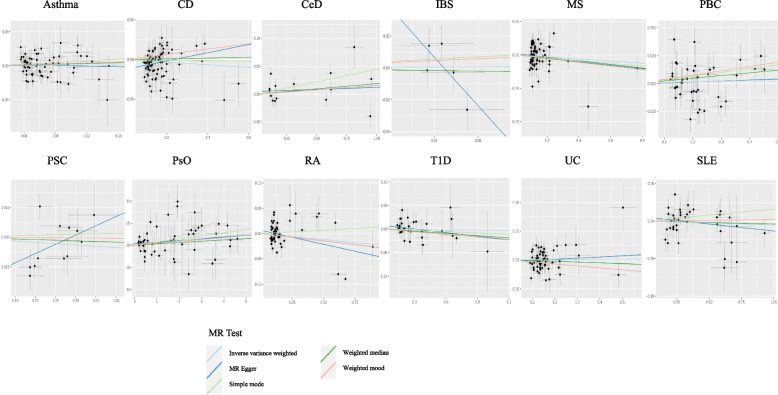
Scatter plots showing the MR effect of each exposure on ALS. The *x*-axis represents the genetic association with autoimmune disease risk; the *y*-axis represents the genetic association with the risk of ALS. Each line represents a different MR method. CD, Crohn’s disease; CeD, celiac disease; IBS, irritable bowel syndrome; MS, multiple sclerosis; PBC, primary biliary cirrhosis; PSC, primary sclerosing cholangitis; PsO, psoriasis; RA, rheumatoid arthritis; T1D, type 1 diabetes; UC, ulcerative colitis; SLE, systemic lupus erythematosus; MR, Mendelian randomization. No significant associations were detected

Our IVW results showed that liability to autoimmune disorders does not affect ALS risk which is consistent with results from the other MR methods including MR Egger and weighted median (Table 2, Fig. 1). The results of the MR analyses investigating the causal relationship between ALS and 12 autoimmune disorders are shown in Table 2. The estimates (ORs) represent the effects on ALS risk of genetically predicted liability to each disease exposure. Our MR analyses had 80% power to detect the effect sizes of liability to exposures on ALS corresponding to OR from 1.045 to 2 in the main MR analyses and the effects of liability to ALS on the risk of autoimmune diseases corresponding to OR from 1.32 to 2.37 in the reverse MR analyses (Table 1). The variance in the exposures explained by their respective set of SNPs ranged from 0.08 to 31% (Tables 2 and 3). All instruments had an *F*-statistics of > 39, which is above the standard cutoff (> 10) indicating sufficient instrumental strength [50, 51] (Tables 2 and 3). Applying Bonferroni correction for multiple testing, a *p*-value below 2.1E−03 was considered as significant.

**Table 2:** Results of the MR analyses between liability to autoimmune disorders and the ALS risk

Exposure	N SNPs	*r*^2^	*F*-statistics	Inverse variance weighted			Weighted median			MR Egger		
				OR	CI	pval	OR	CI	pval	OR	CI	pval
Asthma	67	0.002	71.785	1.014	0.964–1.066	0.599	1.031	0.955–1.113	0.437	0.988	0.862–1.132	0.859
CD	76	0.010	96.324	0.984	0.963–1.005	0.124	1.005	0.977–1.033	0.750	1.053	0.997–1.113	0.067
CeD	15	0.057	333.990	1.016	0.992–1.040	0.185	1.019	0.995–1.044	0.122	1.007	0.972–1.044	0.701
IBS	5	0.001	38.970	1.013	0.727–1.412	0.940	0.960	0.667–1.384	0.825	0.047	0.002–1.403	0.176
MS	68	0.009	69.687	0.985	0.962–1.008	0.166	0.974	0.943–1.007	0.666	1.027	0.977–1.080	0.750
PBC	40	0.018	92.080	1.007	0.984–1.031	0.547	1.024	0.995–1.055	0.104	1.009	0.943–1.080	0.795
PSC	12	0.021	42.303	0.978	0.934–1.024	0.349	0.990	0.944–1.38	0.677	1.169	0.940–1.454	0.192
PsO	53	0.310	58.132	1.005	1.001–1.009	0.012	1.003	0.997–1.009	0.312	1.004	0.999–1.009	0.132
RA	51	0.010	64.953	0.976	0.945–1.008	0.833	0.971	0.928–1.015	0.976	0.946	0.898–0.997	0.373
T1D	32	0.021	71.107	0.998	0.978–1.018	0.830	0.984	0.955–1.015	0.312	1.026	0.986–1.067	0.219
UC	54	0.008	72.544	1.002	0.975–1.028	0.911	0.987	0.954–1.022	0.481	1.021	0.938–1.110	0.637
SLE	39	0.034	117.386	0.995	0.980–1.010	0.509	0.996	0.977–1.016	0.694	0.981	0.950–1.014	0.269

The ORs express effects of liability to each exposure on ALS risk

*OR* odds ratio, *CI* confidence interval, *MR* Mendelian randomization, *SNP* single nucleotide polymorphism, *r*^*2*^ proportion of variance in exposure variable explained by SNPs, *F-statistics* “strength” of the instrumental variable, *CD* Crohn’s disease, *CeD* celiac disease, *IBS* irritable bowel syndrome, *MS* multiple sclerosis, *PBC* primary biliary cirrhosis, *PSC* primary sclerosing cholangitis, *PsO* psoriasis, *RA* rheumatoid arthritis, *T1D* type 1 diabetes, *UC* ulcerative colitis, *SLE* systemic lupus erythematosus

**Table 3:** Results of reverse MR analysis between liability to ALS and risk of autoimmune disorders

Outcome	N SNPs	*N*, proxy SNPs	*r*2	*F*-statistics	Inverse variance weighted			Weighted median			MR Egger		
					OR	CI	pval	OR	CI	pval	OR	CI	pval
Asthma	10	0	0.004	63.343	1.016	0.959–1.077	0.590	1.032	0.969–1.098	0.340	0.983	0.853–1.132	0.817
CD	10	0	0.004	63.343	1.066	0.951–1.194	0.270	1.078	0.927–1.253	0.331	1.200	0.920–1.566	0.215
CeD	2	0	0.023	139.698	2.351	1.167–4.735	0.017	NA	NA	NA	NA	NA	NA
IBS	10	0	0.004	63.343	0.999	0.940–1.062	0.972	0.993	0.925–1.066	0.843	0.966	0.832–1.121	0.272
MS	8	3	0.004	64.947	0.991	0.834–1.178	0.058	1.102	0.933–1.302	0.140	1.254	0.740–2.124	0.267
PBC	6	1	0.004	63.009	1.024	0.816–1.286	0.834	1.028	0.778–1.359	0.838	1.137	0.666–1.941	0.663
PSC	9	0	0.004	65.403	1.244	0.991–1.561	0.059	1.289	0.959–1.733	0.095	1.057	0.575–1.940	0.865
PsO	3	1	0.007	112.585	0.834	0.373–1.867	0.660	0.689	0.428–1.108	0.119	7.243	1.329–39.472	0.262
RA	8	0	0.004	67.835	1.025	0.913–1.151	0.243	1.053	0.910–1.220	0.360	1.173	0.820–1.677	0.941
T1D	10	0	0.004	63.343	0.990	0.811–1.209	0.923	1.148	0.899–1.467	0.271	1.478	0.999–2.188	0.087
UC	10	0	0.004	63.343	1.010	0.862–1.184	0.899	1.046	0.884–1.238	0.604	0.866	0.592–1.266	0.480
SLE	9	0	0.004	65.403	1.197	0.970–1.477	0.093	1.243	0.943–1.638	0.093	1.245	0.726–2.134	0.451

The ORs are effects of liability to ALS on the risk of autoimmune disorders

*CI* confidence interval, *MR* Mendelian randomization, *SNP* single nucleotide polymorphism, *r*^*2*^ proportion of variance in exposure variable explained by SNPs, *F-statistics* “strength” of the instrumental variable, *proxy SNPs* SNPs that are LD “proxies”, *CD* Crohn’s disease, *CeD* celiac disease, *IBS* irritable bowel syndrome, *MS* multiple sclerosis, *PBC* primary biliary cirrhosis, *PSC* primary sclerosing cholangitis, *PsO* psoriasis, *RA* rheumatoid arthritis, *T1D* type 1 diabetes, *UC* ulcerative colitis, *SLE* systemic lupus erythematosus

Sensitivity analyses were performed to detect the presence of horizontal pleiotropy. Significant heterogeneity was apparent in our IVs for RA (MR Egger, *Q p*-value = 6.8E−05; IVW, *Q p*-value = 3.2E−05) which is illustrated in our funnel plot and LOO plot (Additional file 1: Table S1, Additional file 1: Fig. S1). The MR-Egger intercept did not provide evidence for unbalanced horizontal pleiotropy as it was centered around zero for all MR analyses. However, MR-PRESSO identified outlier SNPs for RA (rs9275183), CD (rs42861), and CeD (rs13198474) (Additional file 1: Table S1, Additional file 1: Fig. S1). The distortion test did not suggest significant changes in the effect estimates after removing these outlier SNPs (Additional file 1: Table S1).

Additionally, performing reverse MR studies with liability to ALS as the exposure and risk of the 12 autoimmune diseases as outcomes, we found no significant association. In these studies, we used 2–10 ALS-associated SNPs including proxy SNPs as IVs, which explained 0.004–0.007 of the variances in ALS risk and had an *F*-statistic of more than 63. Significant heterogeneity was apparent in our IVs for ALS (when CeD is the outcome) (IVW, *Q p*-value = 4E−07). However, because of not enough instrumental variables for the MR of ALS and CeD, MR-PRESSO analyses and LOO plots were not possible to be provided. The results of all three MR methods were consistent (Table 3, Additional file 1: Fig. S2).

Using 68 and 44 SNPs robustly and independently associated with CD and UC, respectively, multivariable MR provided evidence that liability to these autoimmune traits does not affect ALS risk.

## Discussion

In this study, we performed a comprehensive bidirectional two-sample MR study to investigate the causal relationships between liability to various autoimmune disorders and ALS risk. Using this approach, we found no relationship between liability to these disorders and ALS, which implies that the reported epidemiological associations could be the result of unmeasured confounding or shared genetic architecture. To distinguish between a true negative result and a lack of validity of the MR studies, multiple sensitivity analyses were applied to ensure that the three MR assumptions were satisfied. Given the consistency of our MR findings across these different methods, we are confident about the validity of our MR analyses to exclude moderate to large causal effects of the exposures on the outcomes.

Our results oppose with an observational study by Turner et al. supporting an association between liability to autoimmune disorders and increased risk of ALS [17]. In another comprehensive study investigating the genetic correlation between ALS and 10 autoimmune disorders, Li et al. reported a positive genetic correlation between ALS and CeD, MS, RA, and SLE, which is in the same direction as those found in a study by Turner et al. [52]. One possible explanation for the described associations between autoimmune disorders and ALS in the absence of causal effect in our MR studies is pleiotropy. In the study by Li et al., using conjunctional FDR statistics, the authors identified shared genetic loci between ALS and autoimmune disorders; these loci encompassed membrane trafficking, vesicle-mediated transport, endoplasmic reticulum (ER) to Golgi anterograde transport, and transport to the Golgi and subsequent modification [52]. These results support the hypothesis that the pathology of ALS might be mediated by a dysfunction of the immune system. However, these findings do not imply that liability to these autoimmune disorders predisposes to ALS and cannot resolve the previous debate on whether dysregulated immunity is the cause of ALS or a consequence of it. Overall, these findings support a shared genetic architecture between ALS and autoimmune disorders, but it is likely that there is no causality between the autoimmune disorders and ALS based on our MR study.

Our study has several limitations. This is a European-based study, and our findings cannot be generalized to other populations. Additionally, some of our MR analyses did not have enough power to detect small effects, due to the limited variance of the exposures explained by the SNP instruments or the limited sample sizes of the outcome GWAS. In this direction, excluding ambiguous or palindromic SNPs from our MR instruments might have further affected the power of our MR studies. Larger GWAS on these autoimmune traits will boost the power of future MR studies to detect associations.

## Conclusions

Our results do not support that liability to various autoimmune disorders affects ALS risk in Europeans and suggest that the observed associations could be a result of shared genetic effects or environmental confounders.

## Supplementary Information

**Additional file 1: Figure S1.** Funnel plots and LOO plots which detect outlier SNPs in RA and CD. **Figure S2.** Scatter plots showing the effect of liability to ALS on risk of autoimmune disorders. **Table S1.** Sensitivity analysis, heterogeneity, and pleiotropy, investigating MR assumption violation. **Table S2.** Sensitivity analysis, heterogeneity, and pleiotropy, investigating MR reverse assumption violation. **Code used in this study **includes all the code used to produce all the results in this paper.

## Data Availability

All data generated and codes used in the current study are available in this published article and Additional file 1 associated with it.

## Abbreviations

ALS: Amyotrophic lateral sclerosis
CD: Crohn’s disease
CeD: Celiac disease
ER: Endoplasmic reticulum
GWAS: Genome-wide association studies
HLA: Human leukocyte antigen
IBS: Irritable bowel syndrome
IVs: Instrumental variables
IVW: Inverse-variance weighted
LOO: Leave-one-out analyses
MR: Mendelian randomization
MS: Multiple sclerosis
OR: Odds ratio
PBC: Primary biliary cirrhosis
PSC: Primary sclerosing cholangitis
PsO: Pspsoriasis
RA: Rheumatoid arthritis
SLE: Systemic lupus erythematosus
SNPs: Single nucleotide polymorphisms
T1D: Type 1 diabetes
UC: Ulcerative colitis
WM: Weighted median

## References

[1] Brown RH, Al-Chalabi A (2017). Amyotrophic lateral sclerosis. N Engl J Med.

[2] Taylor JP, Brown RH, Cleveland DW (2016). Decoding ALS: from genes to mechanism. Nature..

[3] Chio A, Logroscino G, Hardiman O, Swingler R, Mitchell D, Beghi E (2009). Prognostic factors in ALS: a critical review. Amyotroph Lateral Scler.

[4] Gladman M, Zinman L (2015). The economic impact of amyotrophic lateral sclerosis: a systematic review. Expert Rev Pharmacoecon Outcomes Res.

[5] Cooper-Knock J, Jenkins T, Shaw PJ (2013). editors. Clinical and molecular aspects of motor neuron disease. Colloq Ser Genomic Mol Med.

[6] Ryan M, Heverin M, McLaughlin RL, Hardiman O (2019). Lifetime risk and heritability of amyotrophic lateral sclerosis. JAMA Neurol.

[7] Trabjerg BB, Garton FC, van Rheenen W, Fang F, Henderson RD, Mortensen PB, et al. ALS in Danish Registries: Heritability and links to psychiatric and cardiovascular disorders. Neurol Genet. 2020;6(2). 10.1212/NXG.0000000000000398 PMC7073454 32211514

[8] Zhang R, Hadlock KG, Do H, Yu S, Honrada R, Champion S (2011). Gene expression profiling in peripheral blood mononuclear cells from patients with sporadic amyotrophic lateral sclerosis (sALS). J Neuroimmunol.

[9] Zhang R, Gascon R, Miller RG, Gelinas DF, Mass J, Hadlock K (2005). Evidence for systemic immune system alterations in sporadic amyotrophic lateral sclerosis (sALS). J Neuroimmunol.

[10] Prinz M, Priller J (2017). The role of peripheral immune cells in the CNS in steady state and disease. Nat Neurosci.

[11] Murdock BJ, Zhou T, Kashlan SR, Little RJ, Goutman SA, Feldman EL (2017). Correlation of peripheral immunity with rapid amyotrophic lateral sclerosis progression. JAMA Neurol.

[12] Mccombe PA, Henderson RD (2011). The role of immune and inflammatory mechanisms in ALS. Curr Mol Med.

[13] Kawamata T, Akiyama H, Yamada T, McGeer P (1992). Immunologic reactions in amyotrophic lateral sclerosis brain and spinal cord tissue. Am J Clin Pathol.

[14] Henkel JS, Engelhardt JI, Siklós L, Simpson EP, Kim SH, Pan T (2004). Presence of dendritic cells, MCP-1, and activated microglia/macrophages in amyotrophic lateral sclerosis spinal cord tissue. Ann Neurol.

[15] Henkel JS, Beers DR, Wen S, Rivera AL, Toennis KM, Appel JE (2013). Regulatory T-lymphocytes mediate amyotrophic lateral sclerosis progression and survival. EMBO Mol Med.

[16] Duarte F, Binet S, Lacomblez L, Bouche P, Preud’homme J-L, Meininger V (1991). Quantitative analysis of monoclonal immunoglobulins in serum of patients with amyotrophic lateral sclerosis. J Neurol Sci.

[17] Turner MR, Goldacre R, Ramagopalan S, Talbot K, Goldacre MJ (2013). Autoimmune disease preceding amyotrophic lateral sclerosis: an epidemiologic study. Neurology..

[18] Burgess S, Small DS, Thompson SG (2017). A review of instrumental variable estimators for Mendelian randomization. Stat Methodol.

[19] Davies NM, Holmes MV, Smith GD. Reading Mendelian randomisation studies: a guide, glossary, and checklist for clinicians. BMJ. 2018;362. 10.1136/bmj.k601 PMC6041728 30002074

[20] Buniello A, MacArthur JAL, Cerezo M, Harris LW, Hayhurst J, Malangone C (2019). The NHGRI-EBI GWAS Catalog of published genome-wide association studies, targeted arrays and summary statistics 2019. Nucleic Acids Res.

[21] GWAS Catalog. https://www.ebi.ac.uk/gwas/downloads/summary-statistics. Accessed 20 March 2022.

[22] IEU OpenGWAS project. https://gwas.mrcieu.ac.uk. Accessed 20 March 2022.

[23] van Rheenen W, van der Spek R, Bakker M, van den Berg L, Veldink J, van Vugt J (2021). Common and rare variant association analyses in amyotrophic lateral sclerosis identify 15 risk loci with distinct genetic architectures and neuron-specific biology. Nat Genet.

[24] Project MinE. https://www.projectmine.com. Accessed 20 March 2022.

[25] Valette K, Li Z, Bon-Baret V, Chignon A, Bérubé J-C, Eslami A (2021). Prioritization of candidate causal genes for asthma in susceptibility loci derived from UK Biobank. Commun Biol.

[26] De Lange KM, Moutsianas L, Lee JC, Lamb CA, Luo Y, Kennedy NA (2017). Genome-wide association study implicates immune activation of multiple integrin genes in inflammatory bowel disease. Nat Genet.

[27] Trynka G, Hunt KA, Bockett NA, Romanos J, Mistry V, Szperl A (2011). Dense genotyping identifies and localizes multiple common and rare variant association signals in celiac disease. Nat Genet.

[28] Eijsbouts C, Zheng T, Kennedy NA, Bonfiglio F, Anderson CA, Moutsianas L (2021). Genome-wide analysis of 53,400 people with irritable bowel syndrome highlights shared genetic pathways with mood and anxiety disorders. Nat Genet.

[29] Consortium IMSG, ANZgene, IIBDGC, WTCCC2 (2019). Multiple sclerosis genomic map implicates peripheral immune cells and microglia in susceptibility. Science..

[30] Cordell HJ, Fryett JJ, Ueno K, Darlay R, Aiba Y, Hitomi Y (2021). An international genome-wide meta-analysis of primary biliary cholangitis: novel risk loci and candidate drugs. J Hepatol.

[31] Ji S-G, Juran BD, Mucha S, Folseraas T, Jostins L, Melum E (2017). Genome-wide association study of primary sclerosing cholangitis identifies new risk loci and quantifies the genetic relationship with inflammatory bowel disease. Nat Genet.

[32] Tsoi LC, Spain SL, Knight J, Ellinghaus E, Stuart PE, Capon F (2012). Identification of 15 new psoriasis susceptibility loci highlights the role of innate immunity. Nat Genet.

[33] Okada Y, Wu D, Trynka G, Raj T, Terao C, Ikari K (2014). Genetics of rheumatoid arthritis contributes to biology and drug discovery. Nature..

[34] Forgetta V, Manousaki D, Istomine R, Ross S, Tessier M-C, Marchand L (2020). Rare genetic variants of large effect influence risk of type 1 diabetes. Diabetes..

[35] Bentham J, Morris DL, Graham DSC, Pinder CL, Tombleson P, Behrens TW (2015). Genetic association analyses implicate aberrant regulation of innate and adaptive immunity genes in the pathogenesis of systemic lupus erythematosus. Nat Genet.

[36] Hemani G, Tilling K, Davey SG (2017). Orienting the causal relationship between imprecisely measured traits using GWAS summary data. PLoS Genet.

[37] Hemani G, Zheng J, Elsworth B, Wade KH, Haberland V, Baird D (2018). The MR-Base platform supports systematic causal inference across the human phenome. Elife..

[38] Matzaraki V, Kumar V, Wijmenga C, Zhernakova A (2017). The MHC locus and genetic susceptibility to autoimmune and infectious diseases. Genome Biol.

[39] Burgess S, Thompson SG, Collaboration CCG (2011). Avoiding bias from weak instruments in Mendelian randomization studies. Int J Epidemiol.

[40] Burgess S (2014). Sample size and power calculations in Mendelian randomization with a single instrumental variable and a binary outcome. Int J Epidemiol.

[41] Online sample size and power calculator for Mendelian randomization with a binary outcome. https://sb452.shinyapps.io/power/. Accessed 20 March 2022.

[42] Bowden J, Davey Smith G, Burgess S (2015). Mendelian randomization with invalid instruments: effect estimation and bias detection through Egger regression. Int J Epidemiol.

[43] Burgess S, Butterworth A, Thompson SG (2013). Mendelian randomization analysis with multiple genetic variants using summarized data. Genet Epidemiol.

[44] Burgess S, Bowden J, Fall T, Ingelsson E, Thompson SG (2017). Sensitivity analyses for robust causal inference from Mendelian randomization analyses with multiple genetic variants. Epidemiology.

[45] Bowden J, Davey Smith G, Haycock PC, Burgess S (2016). Consistent estimation in Mendelian randomization with some invalid instruments using a weighted median estimator. Genet Epidemiol.

[46] Bowden J, Del Greco MF, Minelli C, Zhao Q, Lawlor DA, Sheehan NA (2019). Improving the accuracy of two-sample summary-data Mendelian randomization: moving beyond the NOME assumption. Int J Epidemiol.

[47] Verbanck M, Chen C-y, Neale B, Do R (2018). Detection of widespread horizontal pleiotropy in causal relationships inferred from Mendelian randomization between complex traits and diseases. Nat Genet.

[48] McGovern DP, Gardet A, Törkvist L, Goyette P, Essers J, Taylor KD (2010). Genome-wide association identifies multiple ulcerative colitis susceptibility loci. Nat Genet.

[49] Burgess S, Thompson SG (2015). Multivariable Mendelian randomization: the use of pleiotropic genetic variants to estimate causal effects. Am J Epidemiol.

[50] Burgess S, Smith GD, Davies NM, Dudbridge F, Gill D, Glymour MM, et al. Guidelines for performing Mendelian randomization investigations. Wellcome Open Res. 2019;4. 10.12688/wellcomeopenres.15555.1 PMC7384151 32760811

[51] Bowden J, Hemani G, Davey SG (2018). Invited commentary: detecting individual and global horizontal pleiotropy in Mendelian randomization—a job for the humble heterogeneity statistic?. Am J Epidemiol.

[52] Li CY, Yang TM, Ou RW, Wei QQ, Shang HF (2021). Genome-wide genetic links between amyotrophic lateral sclerosis and autoimmune diseases. BMC Med.

