# Supplementary material for: Investigation of the causal relationship between ALS and autoimmune disorders: a Mendelian randomization study
Source: BMC Med. 2022 Nov 2;20:382. doi: 10.1186/s12916-022-02578-9 (PMC9628014; doi:10.1186/s12916-022-02578-9)
Supplement: Supplementary file 1 — Additional file 1: Figure S1. Funnel plots and LOO plots which detect outlier SNPs in RA and CD. Figure S2. Scatter plots showing the effect of liability to ALS on risk of autoimmune disorders. Table S1. Sensitivity analysis, heterogeneity, and pleiotropy, investigating MR assumption violation. Table S2. Sensitivity analysis, heterogeneity, and pleiotropy, investigating MR reverse assumption violation. Code used in this study includes all the code used to produce all the results in this paper. [file 12916_2022_2578_MOESM1_ESM.zip › 12916_2022_2578_MOESM1_ESM.docx]

**Additional file 1**

**Figure S1. a.** Funnel plots and b. LOO plots which detect outliers SNPs in RA and CD


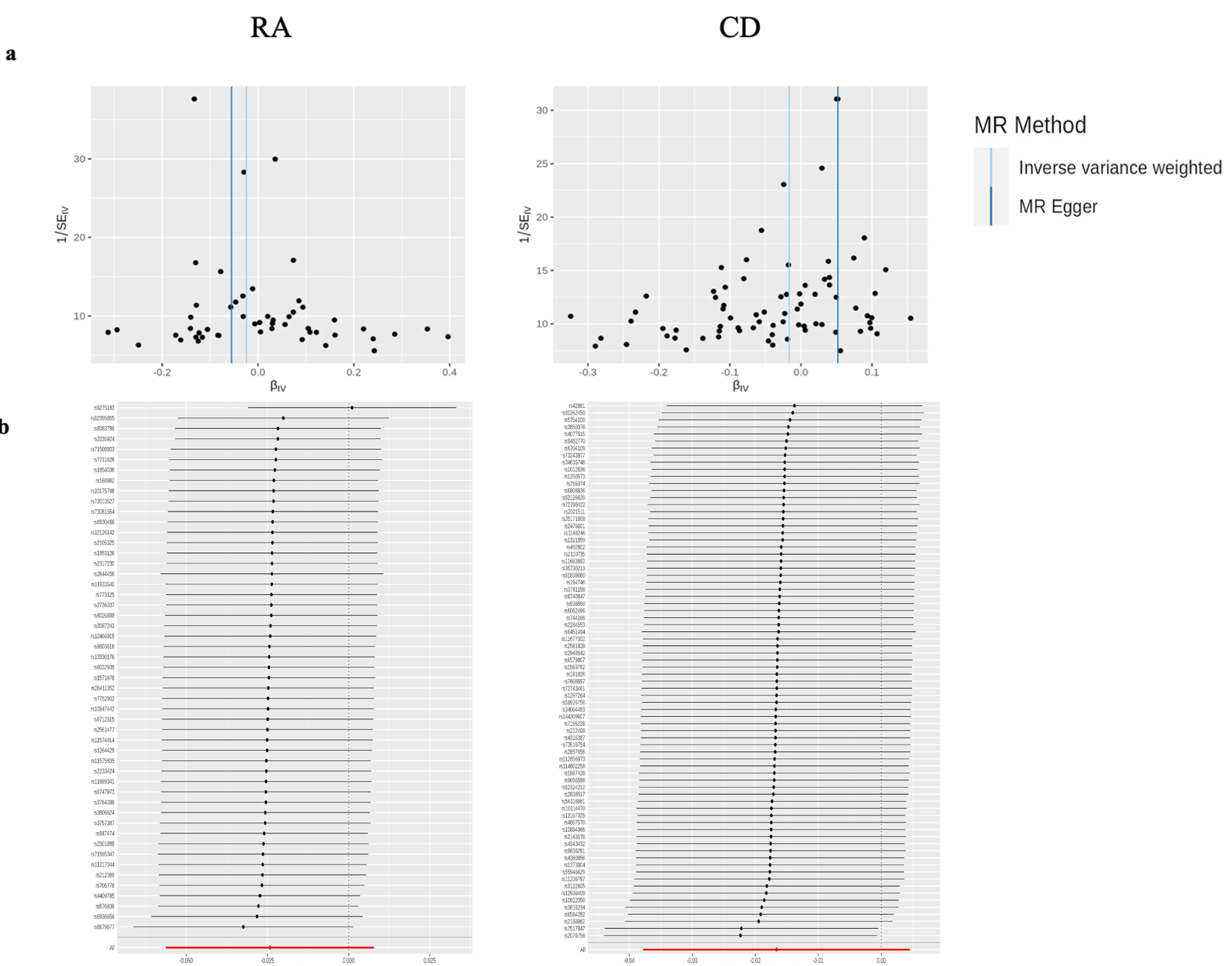


**Figure S2.** Scatter plots showing the effect of liability to ALS on risk of autoimmune disorders.

The x-axis represents the genetic association with ALS risk; the y-axis represents the genetic association with risk of autoimmune disorder. Each line represents a different MR method. CD, Crohn's disease; IBS, Irritable bowel syndrome; MS, Multiple sclerosis; PBC, Primary biliary cirrhosis; PSC, Primary sclerosing cholangitis; PsO, Psoriasis; RA, Rheumatoid arthritis; T1D, Type 1 diabetes; UC, Ulcerative colitis; MR, Mendelian randomization.

**Table S1**. Sensitivity analysis, heterogeneity, and pleiotropy, investigating MR assumption violation

| Exposure | Heterogeneity tests | | | | | | Test for directional horizontal pleiotropy | | | | | | |
| --- | --- | --- | --- | --- | --- | --- | --- | --- | --- | --- | --- | --- | --- |
|  | Inverse variance weighted | | | MR Egger | | | egger_intercept | se | pval | MR-PRESSO global | MR-PRESSO distortion test | MR-PRESSO  Outlier test | |
|  | Q | Q_df | Q_pval | Q | Q_df | Q_pval |  |  |  | pval | pval | pval | Outlier SNPs |
| Asthma | 68.525 | 66 | 3.9E-01 | 68.356 | 65 | 3.6E-01 | 0.002 | 0.005 | 0.690 | 0.392 | NA | NA | NA |
| CD | 109.665 | 75 | 5.6E-03 | 100.208 | 74 | 2.3E-02 | -0.012 | 0.005 | 0.010 | 0.001 | 0.784 | <0.078 | rs42861 |
| CeD | 7.599 | 11 | 0.749 | 7.010 | 10 | 0.725 | 0.011 | 0.014 | 0.460 | 0.744 | NA | NA | NA |
| IBS | 7.136 | 4 | 0.129 | 3.475 | 3 | 0.324 | 0.129 | 0.073 | 0.173 | 0.183 | NA | NA | NA |
| MS | 91.880 | 67 | 0.024 | 91.509 | 66 | 0.021 | 0.002 | 0.004 | 0.606 | 0.021 | NA | 0.070 | NA |
| PBC | 66.920 | 39 | 0.004 | 66.914 | 38 | 0.003 | **4.4E-04** | 0.008 | 0.954 | 0.004 | NA | NA | NA |
| PSC | 22.750 | 11 | 0.019 | 17.973 | 10 | 0.055 | -0.045 | 0.028 | 0.134 | 0.031 | 0.560 | 0.048 | rs10795791 |
| PsO | 51.171 | 52 | 0.506 | 50.551 | 51 | 0.491 | 0.003 | 0.004 | 0.435 | 0.564 | NA | NA | NA |
| RA | 100.342 | 50 | **6.8E-05** | 96.044 | 49 | **3.1E-05** | 0.006 | 0.004 | 0.145 | **<0.001** | 0.007 | <0.051 | rs9275183 |
| T1D | 29.156 | 31 | 0.561 | 27.375 | 30 | 0.604 | 0.007 | 0.005 | 0.192 | 0.527 | NA | NA | NA |
| UC | 67.413 | 53 | 8.8E-02 | 67.135 | 52 | 7.7E-02 | -0.003 | 0.007 | 0.645 | 0.114 | NA | NA | NA |
| SLE | 48.466 | 38 | 0.119 | 47.380 | 37 | 0.118 | 0.005 | 0.006 | 0.363 | 0.135 | NA | NA | NA |

r2, proportion of variance in exposure explained by SNPs; F-statistics, ‘strength’ of the instrumental variable; b, beta; se, standard error, pval, *p*-value; SNP, single nucleotide polymorphism; Q, Cochran’s Q statistic; CD, Crohn's disease; CeD, Celiac disease; IBS, Irritable bowel syndrome; MS, Multiple sclerosis; PBC, Primary biliary cirrhosis; PSC, Primary sclerosing cholangitis; PsO, Psoriasis; RA, Rheumatoid arthritis; T1D, Type 1 diabetes; UC, Ulcerative colitis; SLE, Systemic lupus erythematosus

**Table S2**. Sensitivity analysis, heterogeneity, and pleiotropy, investigating MR reverse assumption violation

| Outcome | Heterogeneity tests | | | | | | Test for directional horizontal pleiotropy | | | | |
| --- | --- | --- | --- | --- | --- | --- | --- | --- | --- | --- | --- |
|  | Inverse variance weighted | | | MR Egger | | | egger_intercept | se | pval | MR-PRESSO global | MR-PRESSO distortion test |
|  | Q | Q_df | Q_pval | Q | Q_df | Q_pval |  |  |  | pval | pval |
| Asthma | 14.968 | 9 | 9.2E-02 | 14.495 | 8 | 0.070 | 0.004 | 0.008 | 0.623 | 0.126 | NA |
| CD | 8.168 | 9 | 0.517 | 7.231 | 8 | 0.512 | -0.015 | 0.015 | 0.361 | 0.519 | NA |
| CeD | 7.267 | 4 | 0.122 | 4.231 | 3 | 0.238 | 0.067 | 0.046 | 0.239 | 0.213 | NA |
| IBS | 13.610 | 9 | 0.137 | 13.195 | 8 | 0.105 | 0.004 | 0.008 | 0.629 | 0.164 | NA |
| MS | 13.114 | 7 | 6.9E-02 | 11.468 | 6 | 7.5E-02 | -0.025 | 0.027 | 0.389 | 0.119 | NA |
| PBC | 2.446 | 5 | 0.785 | 2.269 | 4 | 0.686 | -0.012 | 0.028 | 0.696 | 0.823 | NA |
| PSC | 4.269 | 8 | 0.832 | 3.943 | 7 | 0.786 | 0.018 | 0.032 | 0.586 | 0.851 | NA |
| PsO | 6.733 | 2 | 0.035 | 0.045 | 1 | 0.833 | -0.439 | 0.170 | 0.235 | NA | NA |
| RA | 6.889 | 7 | 0.441 | 6.251 | 6 | 0.396 | -0.015 | 0.019 | 0.464 | 0.458 | NA |
| T1D | 12.789 | 9 | 0.172 | 7.889 | 8 | 0.444 | -0.049 | 0.022 | 0.058 | 0.179 | NA |
| UC | 17.810 | 9 | 0.037 | 16.247 | 8 | 0.039 | 0.019 | 0.021 | 0.406 | 0.063 | NA |
| SLE | 8.064 | 8 | 0.427 | 8.036 | 7 | 0.329 | -0.005 | 0.030 | 0.880 | 0.542 | NA |

r2, proportion of variance in exposure explained by SNPs; F-statistics, ‘strength’ of the instrumental variable; b, beta; se, standard error, pval, *p*-value; SNP, single nucleotide polymorphism; Q, Cochran’s Q statistic; CD, Crohn's disease; CeD, Celiac disease; IBS, Irritable bowel syndrome; MS, Multiple sclerosis; PBC, Primary biliary cirrhosis; PSC, Primary sclerosing cholangitis; PsO, Psoriasis; RA, Rheumatoid arthritis; T1D, Type 1 diabetes; UC, Ulcerative colitis; SLE, Systemic lupus erythematosus

**Code used in this study**

#Select significant p values

awk -F"\t" '{if (NR==1||$8<=5e-08) print $0}' exposure.txt > exposure_sig.txt

This part should not be run in interactive node

#Open R, install packages if necessary

#Read exposure data and perform clumping with standard parameters

library(TwoSampleMR)

exp_data <- read_exposure_data("exposure_sig.txt", sep="\t", snp_col= "SNP", beta_col= "b", eaf_col= "EAF", se_col= "StdErr", effect_allele_col= "A1", other_allele_col= "A2", pval_col= "p", samplesize_col= "SampleSize", ncase_col= "N_cases", ncontrol_col= "N_controls", clump=TRUE)

#Save and quit

write.csv(exp_data,file="exposure_clumped.csv",quote=FALSE)

#This part should be run in interactive node

#Open R, install packages if necessary

library(ggplot2)

library(dplyr)

library(MRPRESSO)

library(TwoSampleMR)

#Read exposure and outcome data

exp_data <- read_exposure_data("exposure_clumped.csv", sep=",", snp_col = "SNP", beta_col = "beta.exposure", eaf_col = "eaf.exposure", se_col="se.exposure", effect_allele_col = "effect_allele.exposure", other_allele_col= "other_allele.exposure", pval_col = "pval.exposure", samplesize_col = "samplesize.exposure", ncase_col= "ncase.exposure", ncontrol_col="ncontrol.exposure", clump=FALSE)

out_data <- read_outcome_data(snps = exp_data$SNP,filename = "outcome.txt", sep="\t",  snp_col = "SNP", beta_col = "b", se_col = "StdErr", eaf_col = "EAF", effect_allele_col = "A1", other_allele_col = "A2", pval_col = "p", ncase_col = "N_cases", ncontrol_col = "N_controls",samplesize_col="SampleSize")

out_data$r.outcome <- get_r_from_lor(out_data$beta.outcome, out_data$eaf.outcome, out_data$ncase.outcome, out_data$ncontrol.outcome, 0.01,  model = "logit")

#To detect if there is any IVs not available in outcome data

missing <- exp_data %>% filter ( !SNP %in% out_data$SNP)

#Read exposure data (first column has to be SNP column)

exposuregwas <- read_table("exposure.txt")

dfEx <- as.data.frame(exposuregwas)

#Read outcome data

outcomegwas <- read_table("outcome.txt")

outcome <- as.data.frame(outcome)

#Find proxy SNP using LDlinkR

library(LDlinkR)

for (i in 1:nrow(missing)) {

x <- LDproxy(missing [i , 1], pop = "EUR", r2d = "r2", token = "6e1ccff03a31")

eligible <- x[x$R2 >= 0.8 , ]

A <- dfEx %>%

filter ( SNP %in% eligible$RS_Number)

Common <- outcome %>% filter ( SNP %in% A$SNP)

R2vals <- eligible %>%

filter( RS_Number %in% Common$SNP)

missing$proxySNP[i] <- R2vals[1 , 1]

}

#missing now contains a column of proxy SNPs to incorporate to exposure data

#Harmonization of data

dat <- harmonise_data(exposure_dat=exp_data, outcome_dat=out_data, action=2)

#To calculate r2 and perform Steiger filtering, we added the columns. Prevalence should also be specified in each exposure and outcome file or manually added

dat$units.outcome<-"log odds"

dat$units.exposure<-"log odds"

dat1<-subset(dat, dat$eaf.exposure!="NA")

dat1$r.exposure <- get_r_from_lor(dat1$beta.exposure, dat1$eaf.exposure, dat1$ncase.exposure, dat1$ncontrol.exposure, 0.1,  model = "logit")

dat1$prevalence.exposure<- 0.1

dat1$prevalence.outcome<- 0.01

#Steiger filtering was performed to exclude SNPs that explain more variance in the outcome than in the exposure

steiger <- steiger_filtering(dat1)

#MR PRESSO to detect horizontal pleiotropy

sig <- subset(steiger, steiger$steiger_dir==TRUE)

presso <-mr_presso(BetaOutcome = "beta.outcome", BetaExposure = "beta.exposure", SdOutcome = "se.outcome", SdExposure = "se.exposure", OUTLIERtest = TRUE, DISTORTIONtest = TRUE, data= sig, NbDistribution = 1000,  SignifThreshold = 0.05)

capture.output(print(presso), file = "presso.txt")

#Perform MR

mr(sig)

#F-statitistics and R2 were calculated for each exposure

#Calculate R2 per SNP

sig$rq=2*(sig$beta.exposure)^2*(sig$eaf.exposure)*(1-sig$eaf.exposure)

#Calculate R2 total

R2<-mean(sig$rq)

capture.output(print(R2), file="r2.txt")

#Calculate a per-SNP F statistic

sig$EAF2 <- (1 - sig$eaf.exposure)

sig$MAF <- pmin(sig$eaf.exposure, sig$EAF2)

PVEfx <- function(BETA, MAF, SE, N){

pve <- (2*(BETA^2)*MAF*(1 - MAF))/

((2*(BETA^2)*MAF*(1 - MAF)) + ((SE^2)*2*N*MAF*(1 - MAF)))

return(pve)

}

N <- sample.size

sig$PVE <- mapply(PVEfx, sig$beta.exposure, sig$MAF, sig$se.exposure, N )

sig$FSTAT <- ((N - 1 - 1)/1)*(sig$PVE/(1 - sig$PVE))

# Calculate a total instrument F statistic

k <- nrow(subset(sig, sig$ambiguous == FALSE))

N <- sample.size

F <- ((N - 1)/k)*((sum(sig$PVE))/(1 - sum(sig$PVE)))

capture.output(print(F), file = "f.txt")

#Save the data

 write.csv(sig,file="sig.csv",quote=FALSE)

#Output from the `mr_report` function will generate a report containing tables and graphs summarizing the results.

mr_report(sig)

#Scatter plot

res<-[mr](https://mrcieu.github.io/TwoSampleMR/reference/mr.html)(sig)

p1<-[mr_scatter_plot](https://mrcieu.github.io/TwoSampleMR/reference/mr_scatter_plot.html)(res, sig)

p1[[1]]

ggsave(p1[[1]], file="scatter.plot.jpg", width=5, height=5)

#Leave-one-out plot

res_loo<-[mr_leaveoneout](https://mrcieu.github.io/TwoSampleMR/reference/mr_leaveoneout.html)(dat)

p3<-[mr_leaveoneout_plot](https://mrcieu.github.io/TwoSampleMR/reference/mr_leaveoneout_plot.html)(res_loo)

p3[[1]]

ggsave(p3[[1]], file="loo.jpg", width=5, height=5)

#Funnel plot

res_single<-[mr_singlesnp](https://mrcieu.github.io/TwoSampleMR/reference/mr_singlesnp.html)(sig)

p4<-[mr_funnel_plot](https://mrcieu.github.io/TwoSampleMR/reference/mr_funnel_plot.html)(res_single)

p4[[1]]

ggsave(p4[[1]], file="funnel.plot.plot.jpg", width=5, height=5)

 #Forest plot

res_single<-mr_singlesnp(sig)

p5<-mr_forest_plot(res_single)

p5[[1]]

ggsave(p5[[1]], file="forest.plot.jpg", width=5, height=5)

#Multivariable MR

library(ggplot2)

library(dplyr)

library(MRPRESSO)

library(TwoSampleMR)

library(readr)

library(MRInstruments)

#Read exposures and outcome

id_exposure <- c("exposure1.txt", "exposure2.txt")

id_outcome <- "outcome.txt"

#Extract and clump significant IVs for 2 different exposures

exposure_dat <- mv_extract_exposures_local(id_exposure, sep ="\t", snp_col ="SNP", beta_col ="b", se_col ="StdErr", eaf_col ="EAF", effect_allele_col ="A1", other_allele_col ="A2", pval_col ="p", ncase_col ="N_cases", ncontrol_col ="N_controls", samplesize_col ="SampleSize", log_pval =FALSE, min_pval =1e-200, pval_threshold =5e-08, clump_r2 =0.001, clump_kb =10000, harmonise_strictness =2)

outcome_dat<-[read_outcome_data](https://mrcieu.github.io/TwoSampleMR/reference/read_outcome_data.html)(snps =exposure_dat$SNP, filename ="outcome.txt", sep ="\t", snp_col ="SNP", beta_col ="b", se_col ="StdErr", effect_allele_col ="A1", other_allele_col ="A2", eaf_col ="a1_EAF", pval_col ="p", samplesize_col ="SampleSize")

mvdat <- mv_harmonise_data(exposure_dat, outcome_dat)

res <- mv_multiple(mvdat)

write.csv(res,file="res.csv",quote=FALSE)
