# Supplementary material for: Investigation of the causal relationship between ALS and autoimmune disorders: a Mendelian randomization study
Source: BMC Med. 2022 Nov 2;20:382. doi: 10.1186/s12916-022-02578-9 (PMC9628014; doi:10.1186/s12916-022-02578-9)
Supplement: Supplementary file 1 — Additional file 1: Figure S1. Funnel plots and LOO plots which detect outlier SNPs in RA and CD. Figure S2. Scatter plots showing the effect of liability to ALS on risk of autoimmune disorders. Table S1. Sensitivity analysis, heterogeneity, and pleiotropy, investigating MR assumption violation. Table S2. Sensitivity analysis, heterogeneity, and pleiotropy, investigating MR reverse assumption violation. Code used in this study includes all the code used to produce all the results in this paper. [file 12916_2022_2578_MOESM1_ESM.zip › Figure S2.pdf]

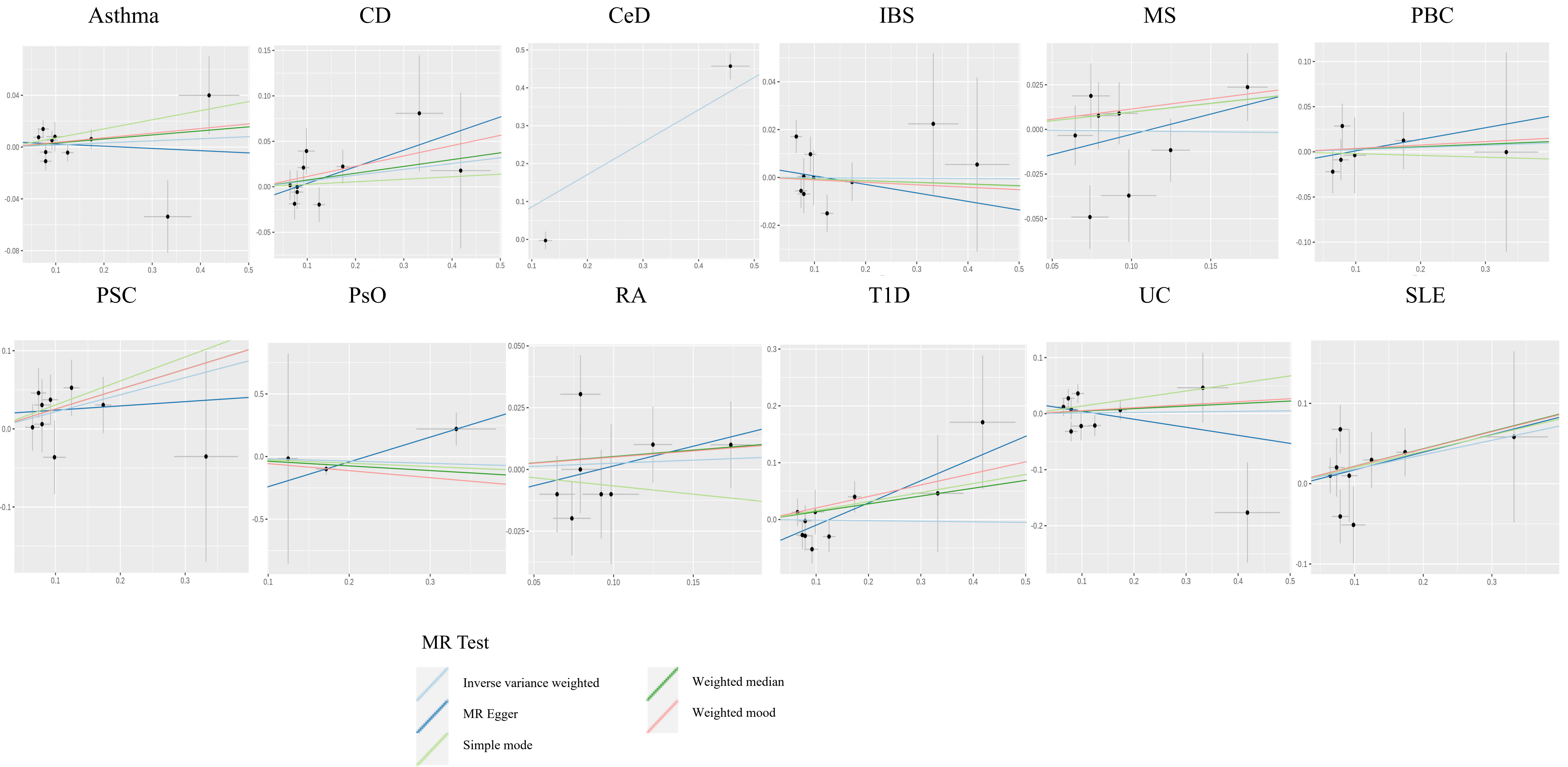
Asthma
CD
CeD
IBS
MS
PBC
PSC
PsO
RA
T1D
UC
SLE
MR Test
Inverse variance weighted
MR Egger
Simple mode
Weighted median
Weighted mood
